# The Anticancer Properties of Tanshinones and the Pharmacological Effects of Their Active Ingredients

**DOI:** 10.3389/fphar.2020.00193

**Published:** 2020-03-19

**Authors:** Li Fu, Bing Han, Yang Zhou, Jie Ren, Wenzhi Cao, Gopal Patel, Guoyin Kai, Jun Zhang

**Affiliations:** ^1^School of Life Sciences, Institute of Plant Biotechnology, Shanghai Normal University, Shanghai, China; ^2^Laboratory of Medicinal Plant Biotechnology, College of Pharmaceutical Science, Zhejiang Chinese Medical University, Hangzhou, China

**Keywords:** *Salvia miltiorrhiza*, tanshinones, autophagy, migration, tumor immunology, apoptosis

## Abstract

Cancer is a common malignant disease worldwide with an increasing mortality in recent years. *Salvia miltiorrhiza*, a well-known traditional Chinese medicine, has been used for the treatment of cardiovascular and cerebrovascular diseases for thousands of years. The liposoluble tanshinones in *S. miltiorrhiza* are important bioactive components and mainly include tanshinone IIA, dihydrodanshinone, tanshinone I, and cryptotanshinone. Previous studies showed that these four tanshinones exhibited distinct inhibitory effects on tumor cells through different molecular mechanisms *in vitro* and *in vivo*. The mechanisms mainly include the inhibition of tumor cell growth, metastasis, invasion, and angiogenesis, apoptosis induction, cell autophagy, and antitumor immunity, and so on. In this review, we describe the latest progress on the antitumor functions and mechanisms of these four tanshinones to provide a deeper understanding of the efficacy. In addition, the important role of tumor immunology is also reviewed.

## Introduction

**FIGURE 1:**
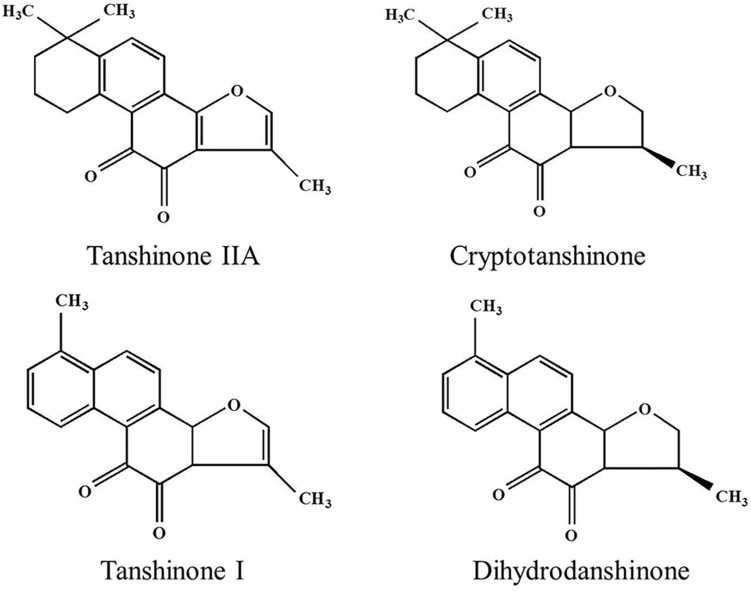
The chemical structure of four tanshinone monomers.

Tanshinone is a natural terpenoid and the main bioactive component isolated from traditional Chinese medicine *Salvia miltiorrhiza.* It is traditionally used in the treatment of cardiovascular and cerebrovascular diseases in China (Chen et al., 2012; Shi et al., 2019; Sun et al., 2019). Modern pharmaceutical studies showed that tanshinone also possess antiangiogenic, antioxidant, antibacterial, anti-inflammatory, and antitumor activities (Wang et al., 2016; Gao et al., 2017; Zhou et al., 2017). According to the chemical structure, tanshinones could be classified into different types, out of which tanshinone IIA (Tan IIA), dihydrotanshinone (DT), tanshinone I (Tan I), and cryptotanshinone (CT) generally considered most important (Hao et al., 2016a; Shi et al., 2016a). Their chemical structures and physicochemical properties are summarized in Figure 1 and Table 1, respectively. The main precursor of tanshinones biosynthesis is geranyl diphosphate (GPP), which is derived from the mevalonate and the 2-C-methyl-d-erythritol-4-phosphate pathway (Kai et al., 2011; Shi et al., 2016b; Cao et al., 2018). A series of downstream enzymes were involved to catalyze the various steps of biosynthesis, and GPP finally transformed into tanshinones (Gao et al., 2009; Guo et al., 2013).

**TABLE 1:** Basic physicochemical properties of tanshinone compounds.

Tanshinones	Molecular formula	Molecular weight	CAS	Detection method	Detection wavelength (nm)	Retention time (min)
Tanshinone IIA	C_19_H_18_O_3_	294.33	568-72-9	HPLC	220	21.44
Dihydrodanshinone	C_18_H_12_O_3_	278.3	87205-99-0	HPLC	280	7.79
Tanshinone I	C_18_H_12_O_3_	276.29	568-73-0	HPLC	220	12.66
Cryptotanshinone	C_19_H_20_O_3_	296.35	35825-57-1	HPLC	220	12.12

*Chromatographic conditions, waters reversed-phase C18 symmetry column; mobile phase, acetonitrile: H_2_O (65:35 vol/vol); temperature, 30°C; flow velocity, 1 mL/min; detection wavelength, 220 nm.*

Tumor is a group of cells/tissues that has lost its control on the normal growth at the gene level due to the activation of various oncogenes or inactivation of tumor suppressors (Noda et al., 1999; Zhang et al., 2013). According to the size and growth characteristics, tumor tissue can be divided into malignant (cancerous) and benign (non-cancerous) (Avgerinou et al., 2017). Malignant tumor grows rapidly and often infiltrate to the surrounding tissues without envelops on the surface. The pathological examination indicated that these cells often exhibit atypical mitosis (Machado et al., 2016). Patients with advanced cancer often exhibited severe systemic symptoms and high recurrence rate after surgical excision, causing a big challenge for cancer therapy (Bax et al., 2016; Bendella et al., 2016; Frohman et al., 2016). In view of the enormous harm caused by cancer, the development of new antitumor treatments has become a research hotspot. Natural products are a good resource of antitumor compounds. In recent years, tanshinones have drawn scientists’ attention because of its broad-spectrum and effective antitumor activities. Their efficacy and the mechanisms have been excavated gradually. This article summarizes the latest researches on the antitumor effects and the mechanisms of the four tanshinones (Tan IIA, DT, Tan I, and CT). Current study may provide reference for the research and development of tanshinone compounds.

## Pharmacological Activities of Different Tanshinone Monomers

Various studies showed that tanshinone compounds have a wide range of pharmacological effects, such as antibacterial, anti-inflammation, antioxidation, and antithrombosis (Zhou et al., 2017; Luo et al., 2019). With the excavating study, people also found the unique effect of tanshinones in the treatment of some diseases, such as diabetic cardiomyopathy (Tao et al., 2019), nephropathy (Liang et al., 2018), and diverse cancers. Here we mainly summarized the antitumor effects of four tanshinone monomers; detailed description is as follows:

### The Antitumor Function and Mechanism of Tan IIA

**FIGURE 2:**
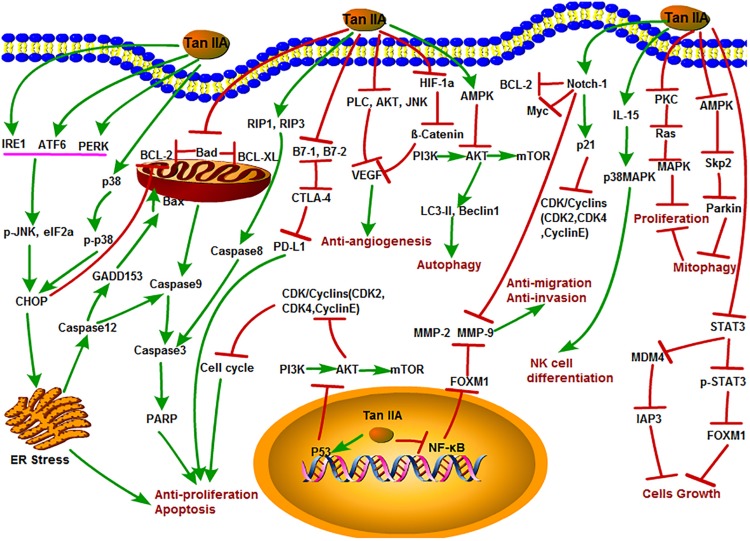
The antitumor mechanism of Tan IIA. Tanshinone IIA inhibits cancer cell proliferation, induces differentiation and apoptosis through MAPK, AMPK/Skp2/Parkin, ER stress and mitochondrial pathway, inhibits of invasion and migration through Notch1/NF-κB signaling, induces of autophagy in cancer cells through PI3K/Akt/mTOR signaling pathway, and inhibits angiogenesis by inhibiting the activity of VEGF. The arrow represents the promotion effect, and the T area represents the inhibition effect.

Tanshinone IIA appears as a kind of red crystalline substance that isolated from *S. miltiorrhiza*. Modern researches showed that Tan IIA has various physiological functions with extremely high medicinal value such as repair on myocardial damage, improvement of microcirculation, and extensive antitumor activity (Lin et al., 2006; Wang J. et al., 2013; Wang et al., 2014; Yang X. J. et al., 2018; Zhang Y. et al., 2018). Meanwhile, as one of the most severely studied monomers in *S. miltiorrhiza*, the antitumor activities and functions of Tan IIA have drawn more and more scientists’ attention. The various antitumor mechanisms of Tan IIA were shown in Figure 2 and discussed in details as follows.

#### Tan IIA Inhibits Tumor Cell Growth and Proliferation

Inhibition of tumor cell growth and proliferation is usually considered as the main strategy of anticancer compounds on diverse tumors. In general, endoplasmic reticulum (ER) stress, apoptosis induction, and cell cycle arrest can inhibit cell proliferation. Previous studies reported that Tan IIA may inhibit the growth and proliferation of various cancer cells, such as lung, breast, liver, leukemia, and colon cancer (Liu et al., 2006; Chen et al., 2012; Zhao et al., 2013; Lin C. Y. et al., 2017; Lu et al., 2018; Wang et al., 2018; Zhou et al., 2018). The ER stress response is usually defined as the unfolded protein response, which is an imbalance between the accumulations of unfolded or misfolded proteins in the ER lumen (Chen W. et al., 2019). Sustained ER stress activates protein interacting with C-kinase (PICK), inositol-requiring enzyme 1 (IRE1), and activating transcription factor 6 (ATF6), leading to the activation of the proapoptotic factor C/EBP homologous protein (CHOP), which further promotes the activation of caspase-dependent apoptosis (Zhu et al., 2019). Previously, Tan IIA increased expression of protein kinase RNA-like ER kinase (PERK), ATF6, inositol-requiring enzyme 1α (IRE1α), caspase-12, and downstream eukaryotic initiation factor 2α (eIF2α), phosphorylated c-Jun N-terminal kinase (p-JNK), and CHOP to activate ER-mediated apoptosis in human pancreatic cancer BxPC-3 cells *in vitro* (Chiu and Su, 2017). Tan IIA also induced ER stress and apoptosis in human breast cancer BT-20 cells by increasing caspase-12, DNA damage-inducible gene 153 (GADD153), caspase-3, p-JNK, phospho-p38 mitogen-activated protein kinases (p-p38), and Bcl-2-Associated X protein (Bax) levels with decreased expression of B-cell lymphoma extra large (Bcl-xl) and p-ERK in a time- and dose-dependent manner (Yan et al., 2012). In another research, Tan IIA increased p53, p21, and Bax; decreased B-cell lymphoma-2 (Bcl-2), cell division cycle gene 2 (cdc2), and cdc25 expression; and induced ER-related apoptosis in hepatocellular carcinoma 15 cells through the regulation of calreticulin, caspase-12, and GADD153 expression (Cheng and Su, 2010). Additionally, Tan IIA inhibited the protective mitophagy through the inhibition of the adenosine monophosphate-activated kinase (AMPK), S-phase kinase–associated protein 2 (Skp2), Parkin pathway, leading to the mitochondria-mediated apoptosis of cancer cells (He and Gu, 2018).

The normal cell cycle circulation is driven by cyclin-dependent serine/threonine kinases and their regulated cyclin subunits. These proteins consist of cyclin-dependent kinases (CDKs), such as CDK2, CDK4, and CDK6, and cyclins, such as cyclin B, cyclin D, and cyclin E (Thangaraj et al., 2019). The mutation and dysregulations of CDKs/cyclins lead to the uncontrolled cell proliferation (Marion et al., 2015). Tan IIA suppressed the growth of breast cancer MCF-7 cell line through arresting the S and G2 phase cell cycle by inhibiting the phosphatidylinositol-3-kinase (PI3K), protein kinase B (Akt), mammalian target of rapamycin (mTOR), protein kinase C (PKC), rheumatoid arthritis (Ras), and mitogen-activated protein kinase (MAPK) signaling pathway. Interestingly, Tan IIA did not function as an Hsp90 inhibitor but could act synergistically with the Hsp90 inhibitors 17-AAG and ganetespib. Tan IIA inhibited the enzymatic activity of PKC especially the PKCζ and PKCε isoforms. Furthermore, the expression of antiapoptosis protein Bcl-2 was decreased, and the levels of cleaved caspase-3 and poly ADP-ribose polymerase (PARP) protein were increased after treatment with a certain concentration of Tan IIA for 24 h (Lv et al., 2018). Similarly, Tan IIA also plays a critical inhibitory role on diverse lung cancer cells. For instance, Tan IIA induced apoptosis and S phase cell cycle arrest in lung cancer PC9 cells by regulating the PI3K-Akt signaling pathway (Liao et al., 2019). Besides, combination of Tan IIA and adriamycin significantly up-regulated the expression of cleaved caspase-3 and Bax and down-regulated the expression of vascular endothelial growth factor (VEGF), VEGFR2, p-PI3K, p-Akt, Bcl-2, and caspase-3; induced apoptosis; and arrested cell cycle at the S and G2 phases in A549 cells (Xie et al., 2016).

The antitumor efficacy of Tan IIA was also found in other malignancies. Signal transducer and activator of transcription 3 (STAT3) is a member of signal-responsive transcription factors and plays a pivotal role in tumorigenesis (Chen et al., 2017; Wang et al., 2017). Activation of the STAT3 signal directly stimulates the expression of forkhead box M1 (FOXM1), which is a regulator of cell cycle (André et al., 2012; Tan et al., 2014). This evidence revealed that STAT3 constitutively activated in gastric cancer. Zhang and his colleagues showed that Tan IIA could suppress gastric cancer cells growth by down-regulating STAT3 and FOXM1 expression (Zhang Y. et al., 2018). Furthermore, Tan IIA could inhibit osteosarcoma MG-63 cell proliferation and achieve its best inhibitory effect with 8.8 mg/L of Tan IIA (Zhang et al., 2012b; Ma et al., 2016a, b). Tan IIA treatment also induced cell apoptosis and arrested cell cycle in human oral cancer KB cells line by mitochondrial pathway via activation of caspase-3, caspase-9, and PARP (Tseng et al., 2014), by MDM4-IAP3 signaling pathway in lung cancer H1299 cells (Zu et al., 2018), and by inducing the formation of cleaved caspase-8 and cleavage of RIP1, RIP3, and MLKL in human hepatocellular carcinoma HepG2 cells (Lin C. Y. et al., 2016).

#### Tan IIA Inhibits Tumor Cell Invasion and Migration

It is well known that the recrudescence after operation and metastasis are the major causes of death in cancer patients. The invasion and migration are two key factors that contributed to the recurrence and metastasis of cancer cells (Lin et al., 2007; Nurnberg et al., 2011). Therefore, effective suppression of tumor invasion and metastasis might be an important part for cancer therapy (Yang et al., 2015). Previous *in vivo* and *in vitro* studies showed that Tan IIA could inhibit the invasion and migration of colon cancer cells (Su and Lin, 2008; Su et al., 2012). High dose of Tan IIA was shown to inhibit astrocytoma migration through up-regulating transmembrane receptor notch homolog 1 (Notch-1) pathway and down-regulating matrix metalloproteinase-9 (MMP-9), cellular-myelocytomatosis viral oncogene (c-Myc), and Bcl-2 expression (Dong W. et al., 2018). FOXM1 is a member of the FOX family and associated with cell fate decisions. Overexpression of FOXM1 promoted tumor progression and metastasis (Tan et al., 2014). Tan IIA could down-regulate FOXM1, MMP-2, and MMP-9 expression in gastric cancer SGC7901 cell line, resulting in the suppression of proliferation and migration in a dose-dependent way (Yu et al., 2017).

#### Tan IIA Inhibits Tumor Angiogenesis

Angiogenesis is a vital step in the physiological process of tissue repair and regeneration, bone remodeling and reproduction, and embryonic development (Folkman, 2006; Garona et al., 2018; Annese et al., 2019). Moreover, it also played a critical role in the progression of tumor formation, infiltration, invasion, and metastasis (Carmeliet, 2006; Seano et al., 2014; Lii et al., 2016; Ao et al., 2017; Yang et al., 2017). Thus, inhibition of angiogenesis has become an effective strategy for cancer therapy. VEGF is a key factor in the production and release of angiogenesis in tumor tissues under hypoxia. There are eight members of the gene family: VEGF-A, VEGF-B, VEGF-C, VEGF-D, VEGF-E, VEGF-F, and placenta growth factor-1 (PIGF-1) and PIGF-2. It has many functions, including stimulating angiogenesis, recruiting new blood vessels, inflammation, and vascular permeability through angiogenic, which constitutes the most important signal pathway in tumor angiogenesis (Xie et al., 2017). In a recent study, Tan IIA dramatically suppressed; VEGF promoted the migration and tube formation of human endothelia progenitor cells through the phospholipase C (PLC), Akt, and JNK signaling pathways without cytotoxic effect (Lee et al., 2017). Meanwhile, Tan IIA was found to effectively restrain β-catenin/VEGF-mediated angiogenesis by targeting transforming growth factor-β (TGF-β1) in normoxic and hypoxia-inducible factor 1α (HIF-1α) in hypoxic microenvironments in human colorectal cancer (Sui et al., 2017). Tan IIA exhibited antiangiogenic effects *in vivo* and *in vitro* by modulating the secretion of MMP-2 and TIMP-2 (Tsai et al., 2011). Other studies also suggested that Tan IIA could inhibit angiogenesis in some cells, such as osteosarcoma cells, breast cancer cells, and vascular endothelial cells (Li et al., 2015; Xing et al., 2015; Huang et al., 2017).

#### Tan IIA Induces Tumor Cell Autophagy

Cell autophagy is an important physiological process in organism development. Basal autophagy is essential to the normal metabolism of cells, which process waste by removing damaged organelles and protein aggregates (Qiu et al., 2017). The latest research focused on autophagy has made great progress in the understanding of the antitumor mechanisms of Tan IIA. Autophagy involves multiple signaling pathways, such as the AMPK and PI3K/Akt/mTOR signaling pathways (Yang et al., 2019; Zhou et al., 2019). AMPK consists of a catalytic subunit (α 1, α2) and two regulatory subunits β (β1 and β2) and γ (γ1, γ2, and γ3). AMPK regulated variety of biochemical pathways, which control the signal of cellular energy metabolism, whereas its dysfunction is associated with many human diseases (Ge et al., 2019). mTOR is present in both mTORC1 and mTORC2 complexes and has a negative regulatory effect on autophagy. PI3K/Akt and AMPK pathways have positive and negative regulation of mTORC1 effect, respectively (Yang et al., 2019).

In another study, Tan IIA induced autophagic cell death viaactivation of AMPK and ERK and inhibition of mTOR and rapamycin(mTOR), ribosomal protein S6 kinase (p70 S6K) in KBM-5 leukemia cells(Yun et al., 2013; Han et al., 2018). Moreover, it was pointed that thesurvival of osteosarcoma cell could be inhibited by Tan IIA throughthe PI3K/AKT signaling pathway, indicating that Tan IIA could effectively induce autophagy in human osteosarcoma cells(Yen et al., 2018). Analogously, Tan IIA induced autophagocytosis of tumor cells by activating autophagic-related Beclin-1 and light chain3-II (LC3-II) expression in melanoma A375 cells (Li et al., 2017). In addition, Tan IIA might induce the autophagy in oral squamous cell carcinoma by both activating the Beclin-1/Atg7/Atg12-Atg5 pathway and inactivating the PI3K/Akt/mTOR pathway (Ye et al., 2018). Another recent study proved that Tan IIA suppressed colorectal cancer cell growth, decreased mitochondrial membrane potential, and inhibited mitophagy through inactivation of AMPK/Skp1/Parkin pathway (He and Gu, 2018).

#### Tan IIA Induces Tumor Immune Checkpoint Blockade

Programmed cell death-ligand 1 (PD-L1) expressed on various cancer cells played a protective role against the cytotoxicity of immune cells. It interacts with programmed cell death-1 receptor (PD-1) to inhibit the cytotoxicity of T cells and block the antitumor immune response. Thus, dismissing immune suppression using immune checkpoint blockade agents is helpful for cancer therapy. Tan IIA was shown to inhibit breast cancer BT-20 cells by inhibiting the expression of PD-L1, cytotoxic T-lymphocyte-associated antigen 4 (CTLA-4), cluster of differentiation 80 [B7-1 (CD80)], and B7-2 (CD86) (Su, 2018). In addition, Tan IIA enhanced interleukin 15 (IL-15)–mediated natural killer (NK) cell differentiation via activation of p38MAPK pathway (Kim et al., 2012).

#### The Antitumor Activities of Tan IIA *in vivo*

To study the antitumor activity and function of a potential anticancer drug, *in vivo* experiments are often necessary and more convincing. In recent years, more and more *in vivo* studies revealed the unique antitumor activity of Tan IIA. Here, the main antitumor studies of Tan IIA *in vivo* are summarized in Table 2. Using Lewis lung cancer mice model, intraperitoneal injection of Tan IIA at 15 mg/kg significantly inhibited tumor growth, neovascularization, and Bcl-2 expression and increased the levels of CD4^+^, CD4^+^/CD8^+^, and NK cells. Moreover, combination of Tan IIA with cyclophosphamide (CTX) showed potent efficacy (Li et al., 2016). Endothelial progenitor cells (EPCs) usually derive from bone marrow that are generally considered as the key regulator in tumor angiogenesis and metastasis (Asahara et al., 1999; Adams et al., 2012; Mund et al., 2012). Tan IIA was first proved to reduce EPC angiogenesis by inhibiting PLC, Akt, and JNK signaling pathways in a chick embryo chorioallantoic membrane model and Matrigel plug assay in mice, indicating that Tan IIA might be the new potential treatment of angiogenesis-related cancers (Lee et al., 2017). In an acute promyelocytic leukemia (APL) NOD/SCID mice model, Tan IIA was found to prolong the survival of APL-bearing mice, prevent APL-mediated body weight reduction, and inhibit the proliferation of APL cells by inducing apoptosis and differentiation (Zhang et al., 2016). Moreover, in NOD-SCID mice xenografted with human osteosarcoma 143B cells, Tan IIA inhibited the expression of CD31 and mitochondrial fusion proteins Mfn1/2 and Opa1, increased the expression of dynamic-related protein 1 (Drp1), induced apoptosis and antiangiogenesis (Huang et al., 2017). In gastric cancer AGS cell xenograft SCID mice model, results showed that treatment with Tan IIA for 8 weeks significantly reduced the protein expression levels of epidermal growth factor receptor (EGFR), inverted gravity flame reactor (IGFR), PI3K, AKT, and mTOR and inhibited AGS cell proliferation by blocking the PI3K/AKT/mTOR pathway (Su and Chiu, 2016). In BxPC-3-derived xenograft tumor model, treatment with Tan IIA induced ER stress by up-regulating the levels of PERK, ATF6, caspase-12, IRE1α, eIF2α, p-JNK, CHOP, and caspase-3 and inhibited the tumor growth *in vivo* (Chiu and Su, 2017).

**TABLE 2:** The antitumor experiment of tanshinone compounds *in vivo*.

Tanshinones	Cancer	Cell line	Treatment concentration	Mechanism	Signal pathway	References
Tan IIA	Breast cancer	MCF-7	30 mg/kg	Proliferation inhibition	PKC↓, Ras/MAPK↓	Lv et al., 2018
Tan IIA	Gastric cancer	SNU-38, MKN1, AGS	25–30 mg/kg	Proliferation inhibition	STAT3↓	Zhang Y. et al., 2018
Tan IIA	Acute promyelocytic leukemia	NB4	10, 100 mg/kg	Proliferation inhibition	–	Zhang et al., 2016
Tan IIA, CT	Lung cancer	Lewis tumor cells	15, 25 mg/kg	Proliferation inhibition	Bcl-2↓, Bax↑	Li et al., 2016
Tan IIA	Osteosarcoma	MG63 cell	2.5–20 mg/kg	Apoptosis and Autophagy induction	Beclin-1↑ LC3II/LC3I↑	Ma et al., 2016a
ATA	Breast cancer	MDA-MB-453	20, 60 mg/kg	Proliferation inhibition	NF-κBp65↓, caspase-3↑	Su et al., 2012
Tan IIA	–	Endothelial progenitor cells	0–10 μM	Angiogenesis inhibition	PLC/Akt↓	Yoder, 2012
Tan IIA	Osteosarcoma	143B cell	20 mg/kg	Autophagy inhibition	HGK-sestrin 2↑ SESN2↑	Yen et al., 2018
Tan IIA	Melanoma	A375	8, 25, 50 mg/kg	Autophagy inhibition	Beclin-1↑ LC3-II↑	Li et al., 2017
Tan IIA	Oral squamous cell carcinoma	SCC-9	50 mg/kg	Autophagy inhibition	Beclin-1↑	Qiu et al., 2017
CT	Liver cancer	Bel-7404	100 mg/kg	Apoptosis induction	p-STAT3↓	Shen et al., 2017
CT	Cholangiocarcinoma	HCCC-9810	10, 25 mg/kg	Cell circle arrest and Apoptosis induction	JAK2/STAT3↓ PI3K/Akt/NFκB↓	Ke et al., 2017a
CT	Glioma	U87	30 mg/kg	Proliferation inhibition	STAT3↓	Cao et al., 2017
DT-I	Hemangioma	Endothelioma (EOMA)	10, 40 mg/kg	Angiogenesis inhibition and Apoptosis induction	PLC↓	Cai et al., 2018

*↓, down-regulated proteins; ↑, up-regulated proteins.*

According to these studies, it can be concluded that Tan IIA is a promising natural product and deserves further study for cancer therapy. The further detailed research on the antitumor mechanism of Tan IIA will have a clearer understanding about the antitumor function, targets, and the whole regulation network of Tan IIA. Based on this, it may provide a new and effective antitumor strategy for the treatment of cancer.

### The Antitumor Function and Mechanism of CT

**FIGURE 3:**
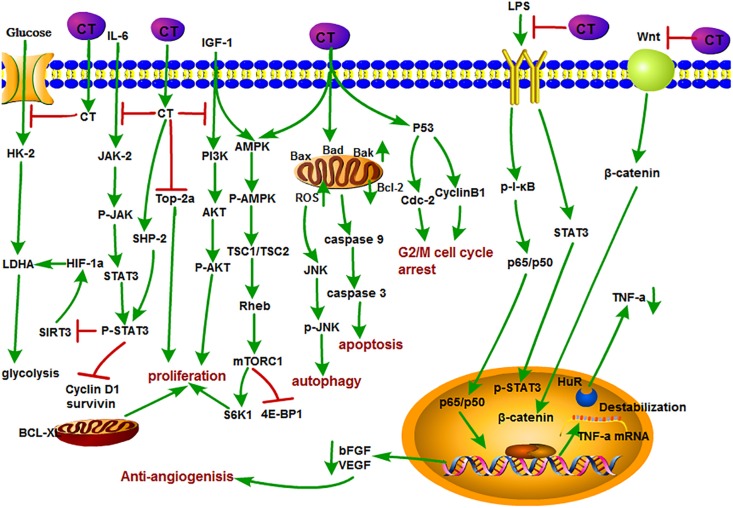
The antitumor mechanism of CT. Cryptotanshinone inhibits the proliferation of cancer cells and induces autophagy and apoptosis by inhibiting IGF-1, IL-6, and glycolysis and mediating the cell cycle and mitochondrial pathway and inhibits angiogenesis by inhibiting the LPS/Wnt signaling pathway. The arrow represents the promotion effect, and the T area represents the inhibition effect.

CT is an important active component in *S. miltiorrhiza* and considered as one of the most effective antineoplastic constituents in tanshinone compounds. Many studies showed that CT significantly inhibited the growth of a variety of tumor cells in addition to its antibacterial and anti-inflammatory activities (Shi et al., 2014; Yu et al., 2014; Hao et al., 2015; Zhou et al., 2016). The antitumor mechanisms of CT are shown in Figure 3. STAT3 is one of the most frequently activated members of the STAT family and plays an important role in the proliferation, survival, invasion, and angiogenesis signaling pathways of various tumors. Abnormal activation of JAK/STAT3 signaling is associated with tumor progression, tumor microenvironment, and immune evasion (Chen et al., 2017; Ke et al., 2017a; Wang et al., 2017). Interleukin 6 is a representative stimulant of STAT3 signaling pathway. CT was identified as a potent STAT3 inhibitor that inhibited the phosphorylation of STAT3 Tyr705 and the target proteins such as survivin, Bcl-XL, and cyclin D1 through blocking the dimerization in DU145 prostate cancer cells (Shin et al., 2009). CT induced apoptosis of esophageal EC109 cancer cells by inhibiting p-STAT3 (Tyr705) and p-JAK2 without effect on the expression of the total STAT3 and JAK2 *in vitro* and *in vivo* (Ji et al., 2019). It is confirming that the antiesophageal cancer effect of CT was associated with the inhibition of IL-6–mediated activation of JAK2/STAT3 signaling pathway (Ji et al., 2019). Similarly, CT was found to have strong inhibition effects on malignant gliomas (MGs) but also preliminarily explored its potential mechanisms through a series of experiments *in vivo* and *in vitro*. In this study, they elaborated that CT could inhibit the proliferation of MG by suppressing the phosphorylation of STAT3 Tyr705 through activating the tyrosine phosphate activity of SHP-2 protein (Lu et al., 2017). Furthermore, CT could significantly suppress the growth and colony-forming of HCCC-9810 and RBE cells by inducing apoptosis in a dose-dependent manner. The underlying mechanism contributed to the inhibition of the JAK2/STAT3 and PI3K/Akt/nuclear factor-κB (NF-κB) pathways. Their work may provide a possible effective treatment for cholangiocarcinoma (Ke et al., 2017b). Aerobic glycolysis is a hallmark of cancer and also called Warburg effect. CT inhibited the expression of glycolysis-related proteins including glucose transporter 1 (GLUT1), hexokinase 2 (HK2), and lactate dehydrogenase A (LDHA) in ovarian cancer Hey cells and xenograft nude mice by repressing STAT3/SIRT3/HIF-1α signaling pathway (Yang Y. et al., 2018). CT inhibited p-STAT5 and p-STAT3, effectively blocked IL-6–mediated STAT3 activation, and reversed chronic myeloid leukemia (CML) fusion gene (BCR-ABL) kinase-independent drug resistance and inhibited key cell coproliferation and drug resistance pathway of K562/ADR in CML (Dong B. et al., 2018). Another study showed that CT induced cell cycle arrest and apoptosis of multidrug-resistant leukemia cell line K562/ADM by inhibiting the expression of cyclin D1, Bcl-2, and eIF4E (Ge et al., 2012). Simultaneously, CT induced autophagic cell death in multidrug-resistant colon cancer cell line SW620 Ad300 via ROS-p38 MAPK–NF-κB signaling pathway (Xu et al., 2017).

Inducing apoptosis of tumor cells is generally considered to be a major mean for the efficacy of antitumor drugs, and it is no exception for CT. For example, CT inhibited the proliferation of human cancer cell A549 and H1299. Detailed studies demonstrated that CT exerted its inhibition effect by down-regulating the insulin-like growth factor 1 receptor (IGF-1R), PI3K/Akt signaling pathway. The insulin-like growth factor 1(bIGF-1) induced IGF-1R and AKT phosphorylation, suggesting that it may become a potential clinical therapeutic agent for the treatment of human lung cancer (Zhang J. et al., 2018). CT induced S-phase cell cycle arrest, apoptosis, and mitochondrial fragmentation in osteosarcoma cells with increased Bax, Bad, and Bak; decreased Bcl-2; and the activation of caspase-3, caspase-8, and caspase-9 expressions. Further Data confirmed that CT directly promoted the interaction of Drp1with Bax, directly, which promoted the translocation of Bax from cytoplasm to mitochondria, resulting in the apoptotic fragmentation of mitochondria (Yen et al., 2019). In a recent study, CT induced apoptosis in melanoma cell lines and increased the sensitivity of A375 cell line to tumor necrosis factor (TNF)–related apoptosis-inducing ligand (TRAIL), which further led to the enhancement of cell death in melanoma cells (Radhika et al., 2018). DNA topoisomerase 2 is an important nuclear enzyme, which regulates cell proliferation by modulating DNA topology and chromatid separation. Treatment with CT dramatically decreased the stabilization of topoisomerase 2a at mRNA level and showed anticancer effect against human prostate cancer *in vitro* and *in vivo* (Kim et al., 2017). In addition, CT also inhibited mTORC1 expression through activating AMPK-TSC2 axis in Rh30 cells (Chen W. et al., 2019). CT induced autophagy by activating JNK signaling through increasing intracellular reactive oxygen species (ROS) formation (Hao et al., 2016b).

As we mentioned earlier, angiogenesis plays a key role in providing oxygen and nutrition for tumor growth and metastasis. Therefore, it has been considered as a potential target for cancer therapy (Yu et al., 2002; Guo et al., 2012; Boreddy and Srivastava, 2013). Studies showed that CT also plays a unique role in this respect. CT inhibited tumor angiogenesis in lipopolysaccharide (LPS)–induced neovascular sprouts in zebrafish embryos and vascularization in mouse Matrigel plug model. Moreover, CT could suppress VEGF-induced tube formation and sprout of human umbilical vein endothelial cells (HUVECs) *in vitro*. Tumor necrosis factor α is generally considered as a key angiogenic factor that is related to both NF-κB and STAT3 pathways. Their further study pointed out that CT could cause the reduction of RNA-binding factor HuR stability and inhibited angiogenesis through posttranscriptional mechanism of TNF-α mediated by NF-κB and STAT3 pathways (Zhu et al., 2016). Basic fibroblast growth factor (bFGF) is a proangiogenic factor that stimulates the migration and spreading of endothelial cell invasion. CT suppressed bFGF-stimulated angiogenesis of bovine aortic endothelial cells *in vitro*. However, Tan IIA showed no effect at the same concentration. CT and Tan IIA have similar structures except C-15 position of dihydrofuran ring. This might infer that the double bond at C-15 position of dihydrofuran ring contributed to the antiangiogenesis efficacy (Hur et al., 2005). In another research, CT inhibited tubular-like structure formation and decreased VEGF expression and LiCl-induced β-catenin augmentation in HUVECs. Thus, CT-mediated antiangiogenesis was associated with the inhibition of Wnt/β-catenin signaling pathway (Chen et al., 2014).

Cryptotanshinone may also exert its anticancer function by enhancing the activity of other antitumor drugs. As we know, arsenic trioxide (ATO) is often used in the treatment of advanced liver cancer. Combined treatment of ATO with CT showed potent inhibition effect on the growth of Bel-7404 cells than ATO or CT alone. Meanwhile, the combination caused the obvious changes in the expression level of antiapoptotic proteins (depressing XIAP, Bcl-2, and survivin) and apoptotic proteins (promoting Bak), which lead to the suppression of tumor growth (Shen et al., 2017). In addition, CT could enhance the sensitivity of ovarian cancer A2780 cells and showed favorable effect on various solid tumors and could sensitize A2780 cells to cisplatin treatment in a dose-dependent manner (Jiang et al., 2017).

**FIGURE 4:**
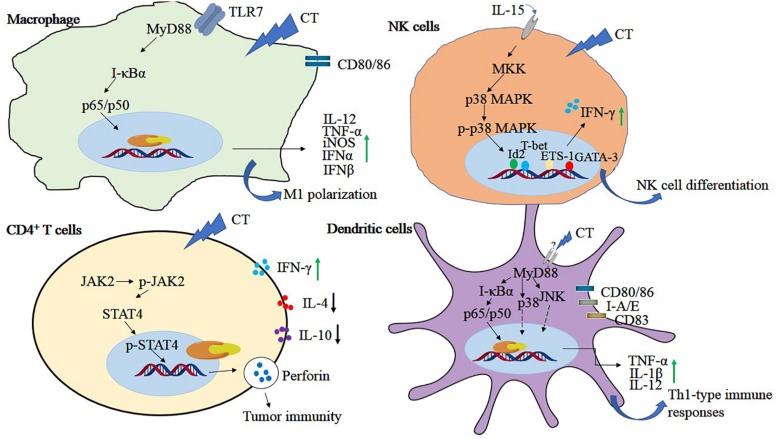
The antitumor immune mechanisms of CT in different cells. Cryptotanshinone plays a role in tumor immunity by regulating NF-κB, MAPK, JAK, and STAT4 signals in immune-related cells, including macrophage, NK cells, CD4^+^T cells, and dendritic cells.

Immunosuppressive tumor microenvironment can lead to tumor-evading immunotherapy, so immunosuppression is a major problem in antitumor therapy. Tumor tissues are infiltrated by immunosuppressive cells such as regulatory T cells and myeloid-derived suppressor cells, and M2-polarized TAMs, which produce inhibitors such as PD-1/PD-L1, lymphocyte activation gene-3, IL-10, and TGF-β, inhibit the proliferation of CD4, CD8 cells, and their immune response (Han et al., 2019; Liu et al., 2019; Yu et al., 2019). CD4^+^ regulatory T cells possess immune functions that are associated with tumor cell immunosuppressive process. CT could increase the cytotoxicity of CD4^+^ T cells without affecting the activity of CD8^+^ T cells. Further investigation indicated that CT activated the JAK 2/STAT 4 pathway in the CD4^+^ T cells, thereby inhibiting the growth of small cell lung cancer (Yong et al., 2016). Perforin is one of the direct target genes of STAT 4 and is activated by IL-12 (Yamamoto et al., 2002). CT might function like IL-12 to activate CD4^+^ T cells to secrete perforin through STAT 4 gene. CT inhibited breast tumor MCF-7 growth by triggering proliferation and increasing perforin secretion of CD4^+^ T cells which is associated with enhancement of the p-JAK 2 and p-STAT 4 expressions. However, the efficacy was abrogated when treated with perforin inhibitor concanamycin, suggesting that CT exhibited the anti–breast tumor activity mainly by activating CD4^+^ T cells to secrete perforin (Zhou et al., 2017). Macrophages are heterogeneous and exert contrast functions. M1 phenotype possess tumor inhibitory properties, whereas tumor-associated macrophages displaying the M2 phenotype promote tumor growth and metastasis (Sica and Bronte, 2007). Moreover, CT was revealed to activate bone marrow–derived macrophages toward an M1 phenotype with elevated expression of CD80, CD86, TNF-α, and IL-12p40 through the TLR7/MyD88/NF-κB and the JAK2/STAT3 signaling pathway in mouse hepatoma Hepa1-6 cells *in vitro*. Furthe*r in vivo* studies in a mouse Hepa1-6 model revealed that CT treatment increased the levels of inducible nitric oxide synthase, TNF-α, interferon α (IFNα), IFNβ, and IL-12p40, but not IL-10 or TGFβ1. Flow cytometry revealed that CT enhanced antitumor T-cell responses with markedly increased infiltration of macrophages, CD45^+^ leukocyte, and CD8^+^ T cells into the tumor tissue. Importantly, combined treatment of CT with anti–PD-L1 successfully eradicated the tumor and showed a synergistic effect on the induction of Hepal-specific immunity responses and developed a long-term antitumor immunity memory. Hepal-bearing mice cured by CT treatment exhibited resistance to Hepal but not EG7 (another C57BL/6 lymphoma cell line) tumor (Han et al., 2019). In addition, CT could promote the maturation of dendritic cells (DCs) and stimulate DC to secrete proinflammatory cytokines TNF-α, IL-1β, and IL-12p70 through the activation of NF-κB, p38, and JNK expression. CT -induced DC maturation is dependent on MyD88 expression because the effects were compromised in MyD88^–/–^ DC. Encouragingly, mice bearing established Lewis lung tumors were cured by CT alone or more effective when combined with anti–PD-L1. Data further demonstrated that CT plus low doses of anti–PD-L1 generated LLC-specific antitumor immune response and immunological memory as resultant tumor-free mice were resistant to rechallenge with LLC, but not B16 melanoma (Liu et al., 2019). Natural killer cells are a subset of lymphocytes crucial for innate and adaptive immune responses that are triggered in the presence of IL-15. CT was shown to increase IL-15–induced NK cell differentiation through enhancing the phosphorylation of p38 MPAK and the expression of transcription factors, such as T-box transcription factor TBX21 (T-bet), GATA-binding protein 3 (GATA-3), inhibitor of DNA biding 2 (Id2), and ETS proto-oncogene 1(ETS-1) (Kim et al., 2012). The antitumor immune mechanisms of CT in different cells are shown in Figure 4.

To sum up, as another lipid-soluble active component of *S. miltiorrhiza*, CT plays an important role in inducing tumor cell apoptosis; inhibiting tumor cell proliferation, invasion, and angiogenesis; and enhancing the activity of other antitumor drugs. However, the study on the antitumor function and mechanism of CT is still in its initial stage, and there are some deficiencies in the analysis of the whole antitumor regulatory network. It is still necessary to perform further studies on CT in terms of molecular biology, cell biology, receptor pharmacology, and so on.

### The Antitumor Function and Mechanism of Tan I

**FIGURE 5:**
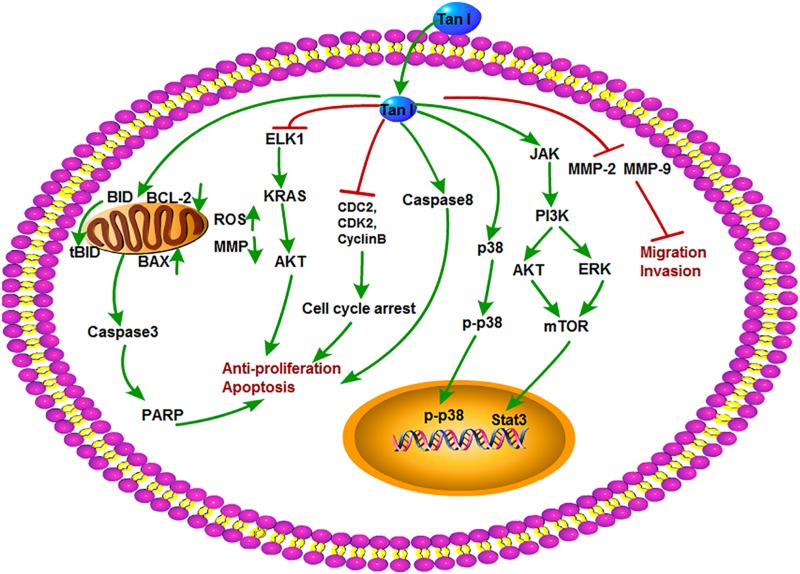
The antitumor mechanism of Tan I. Tanshinone I inhibits proliferation and induces apoptosis of cancer cells by regulating cell cycle and mitochondrial pathway and inhibits cancer cell invasion and migration by inhibiting MMP-2 and MMP-9 signals. The arrow represents the promotion effect, and the T area represents the inhibition effect.

Tanshinone I is a kind of red crystalline powder and accounted for approximately 1.79% of the alcohol extract of *S. miltiorrhiza* roots (Lee et al., 2008). Modern researches showed that Tan I is mainly used in the treatment of cardiovascular and cerebrovascular diseases and also possesses broad-spectrum antitumor activity. Recent studies showed that Tan I significantly inhibited the growth of osteosarcoma cell lines U2OS and MOS-J with IC_50_ (half maximal inhibitory concentration) values around 1 to 1.5 mol/L. It was further shown that Tan I induced apoptosis via up-regulation of Bax and down-regulation of Bcl-2 expression. Tan I also inhibited both the mRNA and protein expression of MMP-2 and MMP-9, which are crucial for tumor metastasis. The underlying mechanism can be concluded as the down-regulation of the JAK/STAT3 signaling pathway (Wang et al., 2019). Similarly, Tan I attenuated proliferation, colony formation, and cisplatin resistance of cervical cancer. The expression of p-AKT and kainate receptors (KARS) was markedly suppressed by Tan I treatment. In contrast, overexpression of KRAS or ETS-like 1 transcription factor (ELK1) markedly impaired the suppression of Tan I on HeLa cells (Dun and Gao, 2018). In the research of Li et al., Tan I showed the most potent effect on the proliferation of lung cancer cells compared with CT and Tan IIA. Tan I also induced apoptosis and G2/M cell cycle arrest *in vitro*; inhibited the expression of Aurora A, survivin, cyclin B, cdc2, and CDK2; and increased the ratio of Bax/Bcl-2. Aurora A–specific siRNA confirmed that Aurora A is a potential target for Tan I. In addition, H1299 xenograft mice oral gavaged with Tan I at a dose of 200 mg/kg exhibited the marked reduction of tumor weight, angiogenesis, and Aurora A expression *in vivo* (Li et al., 2013). Tan I was explored that could cause the death of tumor multidrug resistance cells by inducing PARP, caspase-3, caspase-8, and caspase-9 cleavage and decreasing mitochondria membrane potential without influence on drug transporter proteins P-glycoprotein (P-gp) and multidrug resistance protein 1 (MRP1). It enhanced the depression of p-705-STAT3 and the secondary activation of p38-, AKT-, and ERK-involved signaling networks (Xu et al., 2013). Moreover, Tan I was also proven to induce the apoptosis of prostate cancer cells and enhance its sensibility to TRAIL (Shin et al., 2018). In addition, Tan I could induce apoptosis in both estrogen receptor positive MCF-7 cells and negative MDA-MB-231 cells via the activation of caspase-3 and Bax and the inhibition of Bcl-2 expression (Nizamutdinova et al., 2008). The antitumor mechanisms of Tan I are shown in Figure 5.

### The Antitumor Function and Mechanism of DT

**FIGURE 6:**
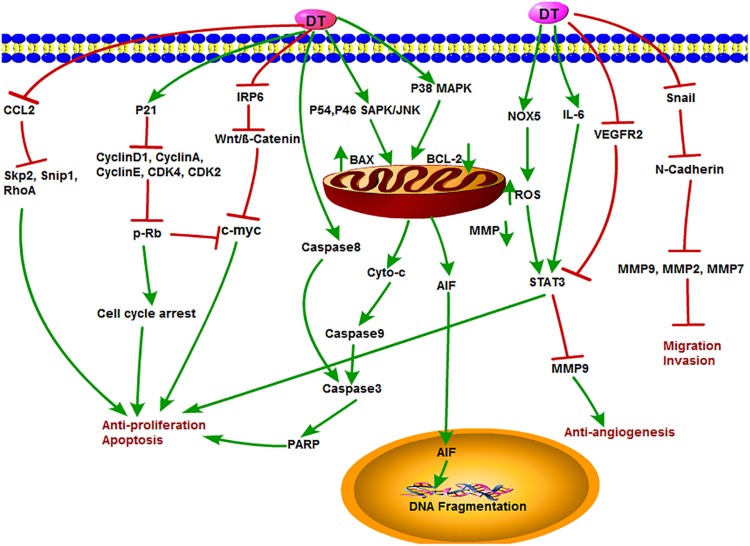
The antitumor mechanism of DT. Dihydrotanshinone inhibits cancer cell proliferation and induces apoptosis by mediating signals such as Wnt/β-Catenin, AMPK/Akt/mTOR, MAPK, CCL2, and p21 and inhibits the invasion and migration of cancer cells and angiogenesis by inhibiting MMP-2, MMP-9, and VEGFR2 signals. The arrow represents the promotion effect, and the T area represents the inhibition effect.

Dihydrotanshinone is also an important component of tanshinone compounds. However, there is still less research on its antitumor functions and mechanisms compared to the other three monomers. DT exhibited special biological inhibitory activities on diverse tumors (Chen X. et al., 2019). The antitumor mechanisms of DT are shown in Figure 6. DT inhibited the growth of liver cancer cells with an EC_50_ (50% effective concentration) value of 2.52 μM. In this study, DT induced caspase-3, -8, and -9 cleavages in a concentration-dependent manner. Moreover, DT significantly induced phosphorylation of P54 and P46 SAPK/JNK (Thr183/TYR185) and p38MAPK (th180/bar182), increased Bax and decreased cytochrome c in mitochondria after p38 MAPK activation, but significantly inhibited PARP cleavage after p38 MPAK inhibition. This indirectly demonstrates that ROS-mediated phosphorylation of p38MAPK is involved in a certain extent in the apoptosis of human hepatoma HepG 2 cells induced by DT (Lee et al., 2009). Cancer stem cells (CSCs) play a key role in tumor metastasis and recurrence. DT activated NOX5 to generate ROS and then phosphorylate STAT3 expression, reduce IL-6 secretion, and induce CSC death (Kim et al., 2019). Similarly, DT exhibited strong cytotoxicity in HCT116 p53(-/-) and HCT116 p53(+/+) colon cancer cells and induced PARP cleavage in a time-dependent manner. They further observed that DT decreased mitochondria membrane potential and stimulated mitochondria to produce ROS, causing a decrease in mitochondrial metabolites and ROS leakage. However, DT-induced apoptosis was inhibited by the ROS scavenger NAC or catalase–PEG alone, confirming that DT impairs mitochondrial function. These results also suggested that DT induced apoptosis of colon cancer cells through a p53-independent but ROS-dependent pathway (Wang L. et al., 2013). In multidrug-resistant colon cancer cell line SW620 Ad300, DT, and CT were shown to induce accumulation of LC3B-II and increase autophagy flux. In addition, the cytotoxic effects of the two tanshinones were independent of p53, suggesting that both DT and CT inhibited the growth of multidrug-resistant colon cancer cells by inducing autophagic cell death in a p53-independent manner (Hu et al., 2015). Recently, DT-I decreased the expression of MMP-9, MMP-2, MMP-7, Snail, and N-cadherin, thus inhibiting the migration and invasion of osteosarcoma cells. Besides, DT-I increased the expression of PARP and caspase-3, decreased the expression of Bcl-2, and induced apoptosis of osteosarcoma cells through mitochondrial pathway. DT I down-regulated the expression of β-catenin, IRP6 (upstream of β-catenin), c-Myc (downstream of β-catenin), and cyclin D1 protein and suppressed Wnt/β-catenin signaling. In an *in vivo* mouse model, DT-I showed inhibited formation of osteosarcoma. These results suggested that DT-I inhibited the proliferation, migration, and invasion and induced apoptosis of osteosarcoma cells *in vivo* and *in vitro* by inhibiting Wnt/β-catenin signaling pathway (Tan et al., 2019). DT activated the activity of caspase-3, caspase-9, PARP, and cytochrome c release; inhibited proliferation of glioma cell; and induced apoptosis in SHG-44 cells (Cao et al., 2017). Moreover, DT had an antiproliferative effect on human hepatocellular carcinoma cells. DT could induce cell cycle arrest of SK-HEP-1 cells at G0/G1 phase, which led to the inhibition of tumor cell growth by down-regulating the expression of cyclin D1, cyclin A, cyclin E, CDK4, CDK2, c-Myc, and retinoblastoma protein (p-Rb) expression with increased expression of the CDK inhibitor p21, suggesting that the antiproliferative activity of DT is related to the regulation of AMPK/AKT/mTOR and MAPK signaling pathways (Hong et al., 2018). In addition, DT could obviously inhibit the angiogenesis of infantile hemangioma by up-regulating several apoptosis-related proteins, such as caspase-3, caspase-8, caspase-9, PARP, AIF, Bax, cytochrome c, and so on (Cai et al., 2018). Lin et al. accessed data from the Taiwan Computerized Insurance Reimbursement Claims Database and used National Health Insurance Research Database (NHIRD) analysis to find out that *S. miltiorrhiza* has a protective effect on colon cancer patients in clinical practice. DT inhibited protein expression of Skp2, Smad nuclear interacting protein 1 (Snip1), and Ras homolog gene family member A (RhoA) and induced apoptosis of HCT116 cells and HT-29 cells by reducing the secretion of CCL2 in macrophages and blocking the recruitment of colon cancer cells. DT treatment also reduced tumor burden in xenograft nude mice (Lin Y. Y. et al., 2017).

### The Antitumor Activities of Other Tanshinone Compounds

There are also many other liposoluble compounds in *S. miltiorrhiza* except those elaborated above, including Tan IIB, isocryptotanshinone, hydroxytanshinone, and so on (Li et al., 2018). These compounds also exhibit antitumor activities on various cancers. For example, isocryptotanshinone was proven as a STAT3 inhibitor that could induce the apoptosis and autophagy of A549 lung cancer cells (Guo et al., 2016). Isocryptotanshinone treatment down-regulated the expression of cell cycle and apoptosis-related proteins cyclin D1, phosphorylated Rb, E2F transcription factor 1 (E2F1), myeloid cell leukemia 1 (Mcl-1), Bcl-2, and survivin expression; inhibited the phosphorylation of STAT3; and induced cell cycle arrest at G1/G0 phase, thereby inhibiting the proliferation of gastric cancer cells. In addition, isocryptotanshinone also inhibited the gastric tumor growth of BALB/c nude mice *in vivo* (Chen et al., 2018). Moreover, isocryptotanshinone down-regulated the expression of Bcl-2 and Bcl-xl proteins; up-regulated the expression of Bax, Bak, PARP, caspase-3, and caspase-9; induced cell cycle arrest at G1 phase; and decreased mitochondrial membrane permeability in MCF-7 cells. Phosphorylation of JNK, ERK, and p38 MAPK was induced by time and concentration dependence, and the MAPK signal was activated to inhibit the proliferation of MCF-7 cells (Zhang et al., 2015). S222 and S439 are derivatives of Tan I that exhibited anti–multidrug resistance and antiangiogenic properties. In a panel of 15 cancer cell lines, S222 and S439 inhibited STAT3 phosphorylation, induced DNA double-strand breaks, blocked cell cycle at G2/M phase, and induced apoptosis (Tian et al., 2018). Acetyl tanshinone IIA (ATA) is a compound obtained by chemical modification of Tan IIA. Acetyl tanshinone IIA induced G1/S phase arrest and apoptosis in HER2-positive MDA-MB-453, SK-BR-3, and BT-47 breast cancer cells by inhibiting receptor tyrosine kinases (RTKs) EGFR/HER2 and downstream survival signaling pathways. Moreover, ATA significantly inhibited tumor growth in athymic MDA-MB-453 xenograft mouse (Guerram et al., 2015). Acetyl tanshinone IIA inhibited the growth of breast cancer cells and the growth of xenografted mouse by inducing the production of ROS and up-regulating the expression of Bax, cytochrome c, and caspase-3 in breast cancer cells (Tian et al., 2010). PTS33, a sodium derivative of CT, could selectively inhibit prostate cancer cells growth by inhibiting the expression of androgen receptor (AR) protein and blocking the expression of AR-regulated genes (Xu et al., 2012). Neo-tanshinlactone, a natural product isolated from *S. miltiorrhiza*, could selectively inhibit the proliferation of estrogen receptor–positive breast cancer cells by inhibiting the synthesis of ESR1 mRNA and down-regulating the transcription of estrogen receptor α (Lin W. et al., 2016). DYZ-2-90 is a novel ring-opening compound modified by neo-tanshinlactone that induced ERK-mediated cell division arrest and apoptosis in human colorectal cancer cells by activating the stress-related JNK pathway (Wang et al., 2012), although there are still few studies on the anticancer effect of these liposoluble monomers. We believe that with the deepening of research, their unique antitumor functional activities will be gradually excavated.

## The Clinical Challenges of Tanshinones

In recent years, there have been more and more studies on the anticancer effects of tanshinones. However, because tanshinones have a high hydrophobicity, it is difficult to prepare an injection, and the absorption is poor at the time of oral administration or injection. Poor bioavailability has been a major challenge for pharmaceutical development. Studies have shown that the structural modification of tanshinone compounds can solve the solubility problem to some extent and improve the limitation of tanshinones in clinical application (Zhang et al., 2012a).

## Conclusion and Perspectives

Traditional Chinese herbal medicine are important sources of antitumor drugs. From their initial use (in the treatment of cardiovascular and cerebrovascular diseases) to the unique antitumor activities, researches on tanshinone compounds have made a great progress. In the last decade, numerous studies have demonstrated the antitumor properties of tanshinones on various tumors both *in vitro* and *in vivo*. Tanshinones exerted a broad range of antitumor functions such as the induction of apoptosis and autophagy; regulation of cell cycle; inhibition of proliferation, invasion, metastasis, and angiogenesis; and enhancement of immunology. It suggests that tanshinones especially Tan IIA and CT may become a potential antitumor agent and provide a new therapeutic strategy for human cancers.

The investigations on the signaling pathways help us to have a better understanding on the antitumor mechanisms of tanshinones. However, process and pathways are usually extremely large and complicated; because of this, our understanding is quite limited. Besides, studies have proven CT having a potent antitumor immune function, whereas there are few reports on other tanshinone compounds. Because of their similar chemical structures, it can be speculated that they may also possess the immunosuppressive effect. However, further investigation is needed. Meanwhile, the *in vivo* antitumor experiments of the four tanshinones are quite few especially for Tan I and DT. Besides, there is still no strong evidence whether it has cytotoxicity on normal cells. This might be a limit for further clinical application. Therefore, more preclinical or clinical trials are also a direction of future research.

## Author Contributions

LF consulted literatures and drafted the manuscript. BH, YZ, JR, WC, and GP participated in manuscript sorting. JZ and GK revised the manuscript.

## Conflict of Interest

The authors declare that the research was conducted in the absence of any commercial or financial relationships that could be construed as a potential conflict of interest.

## Appendix

**Abbreviations:** Akt, protein kinase B; AMPK, adenosine monophosphate-activated kinase; ATA, acetyltanshinone IIA; ATF6, activating transcription factor 6; B7-1 (CD80), cluster of differentiation 80; Bad, Bcl-2-associated death promoter; Bak, Bcl-2 Antagonist Killer; Bax, Bcl-2-Associated X protein; Bcl-2, B-cell lymphoma-2; Bcl-xl, B-cell lymphoma extra large; BCR-ABL, chronic myeloid leukemia (CML) fusion gene; bFGF, basic fibroblast growth factor; cdc2, cell division cycle gene 2; CDKs, cyclin-dependent kinases; CHOP, C/EBP homologous protein; c-Myc, cellular-myelocytomatosis viral oncogene; CT, cryptotanshinone; CTLA-4, cytotoxic T-lymphocyte-associated antigen 4; Drp1, dynamic-related protein 1; DT, dihydrotanshinone; E2F1, E2F transcription factor 1; EGFR, epidermal growth factor receptor; eIF2α, eukaryotic initiation factor 2α; ELK1, ETS-like 1 transcription factor; ETS-1, ETS proto-oncogene 1; FOXM1, forkhead box M1; GADD153, DNA damage-inducible gene 153; GATA-3, GATA binding protein 3; GLUT1, glucose transporter 1; HGK-sestrin 2, HPK1/GCK-like kinase- sestrin 2; HIF-1α, hypoxia-inducible factor 1α; HK2, hexokinase 2; Id2, inhibitor of DNA biding 2; IFNα, Interferon α; IGF-1R, insulin-like growth factor 1 receptor; IGFR, inverted gravity flame reactor; IL-15, interleukin 15; iNOS, inducible nitric oxide synthase; IRE1, inositol-requiring enzyme 1; JAK2, Janus Kinase II; KARS, kainate receptors; LC3-II, light chain 3-II; LDHA, lactate dehydrogenase A; MAPK, mitogen-activated protein kinase; Mcl-1, myeloid cell leukemia 1; MDM4–IAP3, murine double minute 4- inhibitor of apoptosis 3; Mfn1/2, mitochondrial mitofusin proteins; MLKL, mixed-lineage kinase domain-like pseudokinase; MMP-9, matrix metalloproteinase-9; MRP1, multidrug resistance protein 1; mTOR, mammalian target of rapamycin; MyD88, myeloid differentiation protein 88; NF-κB, nuclear factor-b; Notch-1, transmembrane receptor notch homolog 1; Opa1, optic Atrophy 1; p70 S6K, rapamycin (mTOR), ribosomal protein S6 kinase; PARP, poly ADP-ribose polymerase; PD-1, programmed cell death-1 receptor; PD-L1, programmed cell death-ligand 1; PERK, protein kinase RNA-like ER kinase; P-gp, P-glycoprotein; PI3K, phosphatidylinositol-3-kinase; PICK, protein interacting with C-kinase; PIGF, placenta growth factor; p-JNK, phosphorylated c-Jun N-terminal kinase; PKC, protein kinase C; PLC, phospholipase C; p-p38, phospho-p38 mitogen-activated protein kinases; p-Rb, retinoblastoma protein; Ras, rheumatoid arthritis; RhoA, Ras homolog gene family member A; RIP1, receptor interacting protein kinase 1; ROS, reactive oxygen species; RTKs, receptor tyrosine kinases; SAPK, stress-activated protein kinases; SESN2, sestrin 2; Skp2: S–phase kinase–associated protein 2; Snip1, Smad nuclear interacting protein 1; STATs, Signal transducers and activators of transcription; Tan I, tanshinone I; Tan IIA, tanshinone IIA; T-bet, T-box transcription factor TBX21; TGF-β1, transforming growth factor-beta; TLR7, toll-like receptor 7; TRAIL, TNF-related apoptosis-inducing ligand; VEGF, vascular endothelial growth factor; XIAP, X-linked inhibitor of apoptosis.
